# Australian Bat Lyssavirus: Analysis of National Bat Surveillance Data from 2010 to 2016

**DOI:** 10.3390/v13020189

**Published:** 2021-01-27

**Authors:** Rachel Iglesias, Keren Cox-Witton, Hume Field, Lee F. Skerratt, Janine Barrett

**Affiliations:** 1 Australian Government Department of Agriculture, Water and the Environment, Canberra, ACT 2600, Australia; 2 Wildlife Health Australia, Sydney, NSW 2088, Australia; kcox-witton@wildlifehealthaustralia.com.au; 3 EcoHealth Alliance, New York, NY 10018, USA; hume.field@ecohealthalliance.org; 4 School of Veterinary Science, The University of Queensland, Gatton, QLD 4072, Australia; 5 One Health Research Group, Melbourne Veterinary School, University of Melbourne, Parkville, VIC 3010, Australia; l.skerratt@unimelb.edu.au; 6 Queensland Department of Agriculture and Fisheries, Brisbane, QLD 4000, Australia; janine.barrett@daf.qld.gov.au

**Keywords:** Australian bat lyssavirus, Australia, public health, Pteropus, flying-fox, lyssavirus, zoonosis, One Health

## Abstract

Australian bat lyssavirus (ABLV) was first described in 1996 and has been regularly detected in Australian bats since that time. While the virus does not cause population level impacts in bats and has minimal impacts on domestic animals, it does pose a public health risk. For this reason, bats are monitored for ABLV and a national dataset is collated and maintained by Wildlife Health Australia. The 2010–2016 dataset was analysed using logistic regression and time-series analysis to identify predictors of infection status in bats and the factors associated with human exposure to bats. In common with previous passive surveillance studies, we found that little red flying-foxes (*Pteropus scapulatus*) are more likely than other species to be infected with ABLV. In the four Australian mainland species of flying-fox, there are seasonal differences in infection risk that may be associated with reproductive cycles, with summer and autumn the seasons of greatest risk. The risk of human contact was also seasonal, with lower risk in winter. In line with other studies, we found that the circumstances in which the bat is encountered, such as exhibiting abnormal behaviour or being grounded, are risk factors for ABLV infection and human contact and should continue be key components of public health messaging. We also found evidence of biased recording of some types of information, which made interpretation of some findings more challenging. Strengthening of “One Health” linkages between public health and animal health services at the operational level could help overcome these biases in future, and greater harmonisation nationally would increase the value of the dataset.

## 1. Introduction

Australian bat lyssavirus (ABLV) is a member of the *Lyssavirus* genus (family *Rhabdoviridae*), which includes a number of other viruses associated with bats, as well as rabies lyssavirus [1]. ABLV is antigenically distinct from rabies lyssavirus, which is exotic to Australia [2].

Australia was believed to be free of lyssaviruses until 1996, when ABLV was described in a black flying-fox (*Pteropus alecto*) [3]. Subsequently, it was retrospectively identified in a black flying-fox submitted with neurological signs in 1995 [4]. Bats were not routinely tested for lyssaviruses prior to these detections.

Reservoir hosts for ABLV include flying-foxes (*Pteropus* spp.), the larger bats that feed on pollen, nectar and fruit, and the smaller insectivorous bats often called ”microbats”. The virus has been identified in all four mainland flying-fox species—the grey-headed flying-fox (*P. poliocephalus*; GHFF), black flying-fox (*P. alecto*; BFF), little red flying-fox (*P. scapulatus*; LRFF) and spectacled flying-fox (*P. conspicillatus*; SFF)—and one microbat species, the yellow-bellied sheath-tailed bat (*Saccolaimus flaviventris*). Two variants of ABLV are recognised, a pteropid (flying-fox) variant and a yellow-bellied sheath-tailed bat variant [5]. Serological evidence of infection has been found in a range of Australian microbat species [6,7]. All Australian bat species are assumed to be susceptible to ABLV infection, and serological evidence indicates a wide geographical distribution of the virus in Australia [6]. The prevalence of ABLV infection in wild bat populations is estimated to be below 1% [6]. However, the prevalence is greater amongst sick, injured and orphaned bats, which are more likely to contact humans and domestic animals [8].

As with other lyssaviruses, ABLV is transmitted via contamination of a bite, scratch, mucous membrane or broken skin with saliva or neural tissue from an infected bat and causes a progressive fatal neurological disease. Clinical signs in bats include behavioural changes such as abnormal aggression and agitation, and neurological signs such as inability to fly, paralysis or paresis, unusual vocalisation, tremors and seizures [8]. Infected bats may also present with apparent respiratory difficulties [8], or in some cases may appear normal. There have been three cases of ABLV infection in humans in Australia, all with a history of a bite or scratch from a bat [9,10,11,12], and two cases in domestic animals, both horses infected in the same paddock in 2013, probably due to exposure to a single infected bat [13,14]. Transmission to companion animals is considered a potential means by which humans could be exposed to the virus. Clinically normal dogs with a history of exposure to bats have been found to be seropositive [15], although natural ABLV infection has not been documented in dogs or cats. There is currently no evidence of ABLV infection in other wild or domestic mammals.

The disease has no apparent population level impacts on bats, and minimal impacts on domestic animals, but does pose a public health risk. Because of this risk, test results are collated into a national dataset by Wildlife Health Australia (WHA), working with the national Bat Health Focus Group (BHFG) whose members represent government agencies (agriculture, public health and environment), the CSIRO Australian Centre for Disease Preparedness (ACDP, formerly the Australian Animal Health Laboratory), universities, bat carers, and special interest groups such as the Australasian Bat Society and Australian Speleological Federation. WHA coordinates Australia’s national wildlife disease surveillance system through a network of government and private stakeholders [16]. The BHFG, which is part of this network, uses a collaborative One Health approach to consider bat health issues in relation to the broader context of biosecurity, public health, livestock health and environmental impacts.

Most research into ABLV in Australia over the last decade has investigated the nature of human-bat interactions and the community’s understanding of disease risk from bats [17,18,19,20,21]. Targeted ABLV surveillance in wild bats was conducted around 2000 [6] and, more recently, serosurveillance of live microbats has been conducted in Western Australia [7] and southern Australia [22]. Data from bats submitted for ABLV testing was an initial source of information about the ecology of the virus soon after the disease was first identified [8,23] and has also been used to investigate public health risk in Queensland [24,25].

This paper seeks to describe and analyse data from passive surveillance for ABLV in bats in Australia from 1995 to 2016; analytic emphasis is on the period from 2010 to 2016, as the earlier data comprises ABLV positive results only. While there are inherent biases in passive surveillance data, large scale representative studies of ABLV infection in wild bat populations are challenging due to the low prevalence of the disease and the fact that there is no reliable test to diagnose infection in live animals. This analysis aims to investigate predictors of infection status in bats and factors that increase the likelihood of human contact such as a bite or scratch (regardless of the infection status of the bat). This will support public health and biosecurity policies, decision making and communication in relation to ABLV.

## 2. Materials and Methods

### 2.1. Data, Data Sources and Data Collection

Australia is a federated country, comprised of six states and two territories. Animal health surveillance is undertaken by each of the state and territory governments. Surveillance of bats for ABLV occurs for a variety of reasons including contact between a bat and a person (e.g., a bite or scratch); contact between a bat and a pet dog, cat or other animal; a bat has clinical signs consistent with ABLV infection such as neurological signs or unusual behaviour; or a bat is euthanised for welfare reasons. While submission of bats for ABLV testing following contact with people occurs whenever possible in all states, there is variation among states in the degree to which bats are tested for the other reasons stated above. The distribution and abundance of bat species also varies among states, leading to differences in contact rates between people and bats. The dataset consists of the results of testing of individual bats for ABLV from all states and territories in Australia. The bats were tested at government laboratories using one or more of fluorescent antibody test (FAT), polymerase chain reaction (PCR) assay, immunohistochemistry and virus isolation. Definitive diagnosis of ABLV infection requires the post-mortem detection of viral antigens or RNA in brain, other neural tissue or salivary gland [26,27].

Collation of the national data commenced in 2002 when WHA (then the Australian Wildlife Health Network) was established. The data were collated into a web-enabled database, the electronic Wildlife Health Information System (eWHIS). Over time, the database has been customised for improved ABLV data collection. Initially the positive ABLV results were prioritised, but as the organisation grew, resources became available for collection, moderation and reconciliation of both positive and negative results. A fully reconciled dataset from 2010 to 2016 is the focus of the analysis described in this paper. Every eWHIS record was moderated by a WHA staff member and the dataset was reconciled against reports from the ACDP, which is the national animal health reference laboratory.

Data sources include state and territory government biosecurity agencies, the Queensland government health agency, WHA surveillance partners based in zoo wildlife hospitals, veterinary clinics and universities, and Barrett (2004) [8]. Data fields in eWHIS records include event dates, event summary, location the bat was found (nearest suburb or town), species, number dead/affected, presenting syndrome, details of human or animal contact or other presentation, diagnosis, test details, and laboratory name and number.

One of the challenges faced when analysing passively collected data is to distinguish between real effects on risk of exposure to ABLV and anomalous findings that result from the biases in the dataset. In interpreting the findings, we have used appropriate analytical methods, expert judgement and a sound understanding of the operation of the surveillance system, supplemented by insights from surveillance system participants to interpret the data.

### 2.2. Data Manipulation and Analysis

Each observation was mapped to nearest suburb or town, the most precise spatial information collected, using QGIS version 2.0.1 (QGIS.org). For other analyses, the state or territory was used as the spatial unit of interest. The variables considered in this analysis are explained in Table 1. Several of these variables were created based on information from a free-text field: age, sex, reproductive status, human contact, dog contact, the circumstances by which the bat came to human attention (Table 2), the reason that the bat was submitted for testing, the presence of neurological signs and the presence of traumatic injury. This information was not primarily recorded in the database and was not available in all cases.

Season, species, state, sex, age and reproductive status were considered potential risk factors for ABLV infection status. Year was also considered as an indicator for temporal trends in infection risk among tested bats. The circumstances in which the bat was found, the presence of neurological signs or trauma, and recorded contact with a human or pet, were all considered as potential predictors of ABLV infection status. These variables reflect the clinical outcomes and presentations that are likely given what is known about lyssaviruses. All variables were considered potential risk factors for human contact with the bat.

Associations between pairs of explanatory variables were investigated with a chi-squared test or a generalised linear model depending on the data type. Most explanatory variables were not independent, an unsurprising finding given that the data arise from passive surveillance and not random sampling of bat populations. The dataset was restricted to records relating to flying-foxes (*Pteropus* spp.) for statistical modelling, as these species are the largest component of the dataset. The event (one or more bats submitted together) was the unit of interest for analysis of the spatiotemporal factors and species; the individual was the unit of interest for analysis of the remaining factors. Generalised linear models were constructed to investigate associations between the explanatory variables and the outcome variables (ABLV infection status and human contact) using the stats package in R version 3.5 [28] and a logit link function. The likelihood ratio test (lmtest package) [29] was used to assess the significance of each model relative to a model including only the intercept, and fit was assessed using the Hosmer-Lemeshow goodness-of-fit test (ResourceSelection package) [30]. Models were constructed for all possible combinations of the variables, and selection of the best models was informed by the tests of model significance and the Akaike’s information criterion (AIC).

To further investigate temporal variations, the data were aggregated to a monthly count of bats tested, and monthly prevalence among tested bats, and converted to time series objects using the R stats package. Any gaps in the series were filled with a zero count, or zero prevalence. These time series were decomposed using the stats package. This function separates three components from the time series: a trend component, using moving averages; a seasonal component, generated by averaging values for each month across the time series; and a random component, the remainder when the trend and seasonal components are subtracted from the original values in the time series. Equivalent time series were generated for the complete dataset (all submissions, including all bat species) and for several subsets of the data including for the states Queensland, New South Wales, Victoria, and the species BFF, GHFF and LRFF. The raw series and the trend components of the time series of total submissions and prevalence were plotted with the occurrences of human infection with ABLV (February 2013) and two equine cases of ABLV (May 2013) to determine if these events had any influence on submission of bats for testing. Seasonal components of the total submissions and prevalence for each of the flying-fox species were plotted with the timing of mating and birthing of young [31,32] to see if these life history events had any relationship to the seasonal cycles observed.

## 3. Results

**Figure 1:**
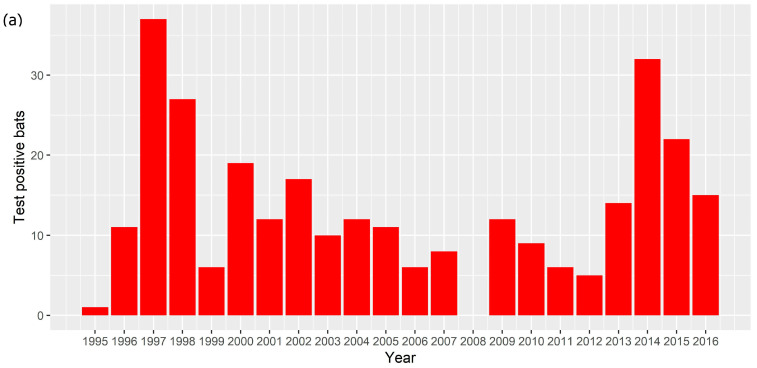

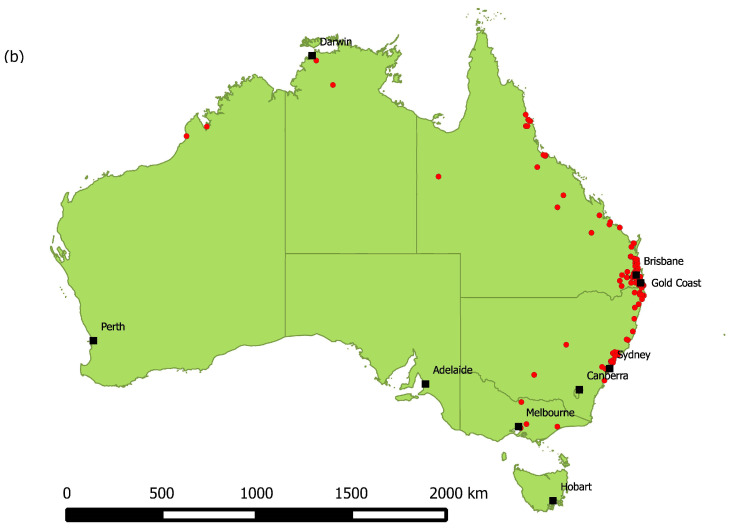
(**a**) Number of ABLV test positive bats per year, 1995–2016. (**b**) Locations from which ABLV test positive bats were submitted, 1995–2016. Red points represent locations at which test positive bats were found. Relevant cities and population centres are marked with a black square.

An ABLV test result was available for 2465 bats. There were 2281 bats tested between 2010 and 2016, of which 103 (4.5%) tested positive, and 2178 (95.5%) tested negative. There were 184 bats that tested positive between 1995 and 2009; the total number tested between 1995 and 2009 is not known. In total between 1995 and 2016, 287 bats tested positive for ABLV. The number of test positive animals varied between 0 and 37 each year (Figure 1a) with a median of 12 and a mean of 13.9. There was only one year (2008) in which no ABLV positive bats were recorded. Test positive bats were detected in all states and territories except Tasmania and the Australian Capital Territory (Figure 1b). Most test positive animals originated from around the major population centres of Brisbane, Sydney and the Gold Coast.

**Figure 2:**
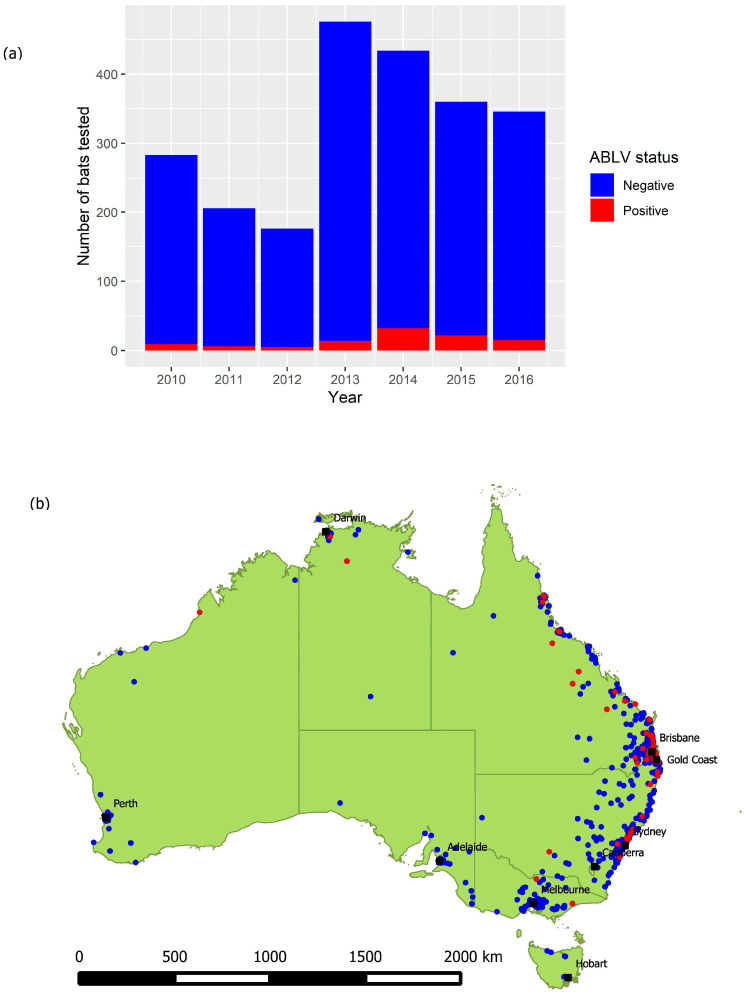
(**a**) Number of bats tested and their ABLV status per year, 2010–2016. (**b**) Locations from which bats were submitted for ABLV testing, 2010–2016. Red points represent locations at which Table 2010. to 2016. BFF comprised 35.9% (820) of the dataset, GHFF comprised 22.8% (521) and LRFF comprised 9.8% (224). SFF made up less than 1% (22) and 6.6% (150) were identified only to genus (*Pteropus* spp.), which based on species distribution and the location found (nearest suburb or town) are most likely to be BFF or GHFF. Other commonly submitted taxa included the following genera of microbats: *Nyctophilus* (5.0%), *Chalinolobus* (3.1%), *Mormopterus* (1.4%), *Vespadelus* (1.4%), *Scotorepens* (1.1%) and *Miniopterus* (1.0%), with 7.1% (163) recorded as an unidentified microbat. The *Mormopterus* genus has undergone a recent taxonomic revision [32]. In this dataset, bats recorded within this genus would now be reclassified as *Ozimops, Setirostris* or *Micronomus.* The most common reasons that bats were tested were pet contact (34.9%) and human contact (33.7%), followed by neurological signs (8.1%). Approximately 20% had a second reason, most commonly trauma (54.9%), non-neurological clinical signs (15.2%) and neurological signs (13.1%). Most submissions came from Queensland (*n* = 1260, 55.2%) or New South Wales (*n* = 469, 20.6%), and very few from Tasmania (*n* = 9, 0.4%).

The total number of bats tested annually between 2010 and 2016 varied from 176 to 476 (Figure 2a), with a median of 346 and a mean of 325.9. Bats have been tested in all states and territories, with greater numbers originating from areas with greater human population density (Figure 2b). Because the dataset does not include test negative animals for the years 1995 to 2009, these years were excluded from subsequent analysis.

While most submissions involved single bats, several types of clustering occurred. In some cases, female bat and dependent young were submitted together (*n* = 15), and occasionally a pregnant female and foetus were both tested (*n* = 4). Sometimes groups of bats were submitted together from a single event (one location and time) (*n* = 41 bats). One large cluster of ABLV infections was recorded in a single town over a period of six months in 2014, resulting in submission of thirteen bats of which eleven tested positive for ABLV. For some factors examined (spatial, temporal, species), the presence of clustering had the potential to introduce bias given that passive surveillance results in sparse sampling, and the clusters may give increased weight to the time, place and species involved. For these factors, the event was taken as the unit of interest for analysis, regardless of the number of bats involved. For events that occurred over several months, the first month was used for temporal analysis, and the event was considered ABLV positive if any of the bats involved tested positive.

### 3.1. Risk Factors for ABLV Infection in Flying-Foxes

Information on sex, age and reproductive status were available for only a proportion of records (29%, 34%, and 3% respectively). On the basis of simple odds ratios (OR), there was no difference in infection risk between males and females (males OR 0.75, 95%CI 0.42–1.34 relative to females); adults and juveniles (juveniles OR 1.36, 95%CI 0.76–2.46 relative to adults); and pregnant (OR 0.57, 95%CI 0.08–1.94) or lactating females and other bats (OR 1.74, 95%CI 0.38–5.34). These variables were not included in the regression models due to the large number of missing values. However, information on demographics was more likely to be available for animals that tested positive for ABLV (OR 2.55 (1.71–3.81) for sex being recorded and 1.72 (1.15–2.56) for age).

**Figure 3:**
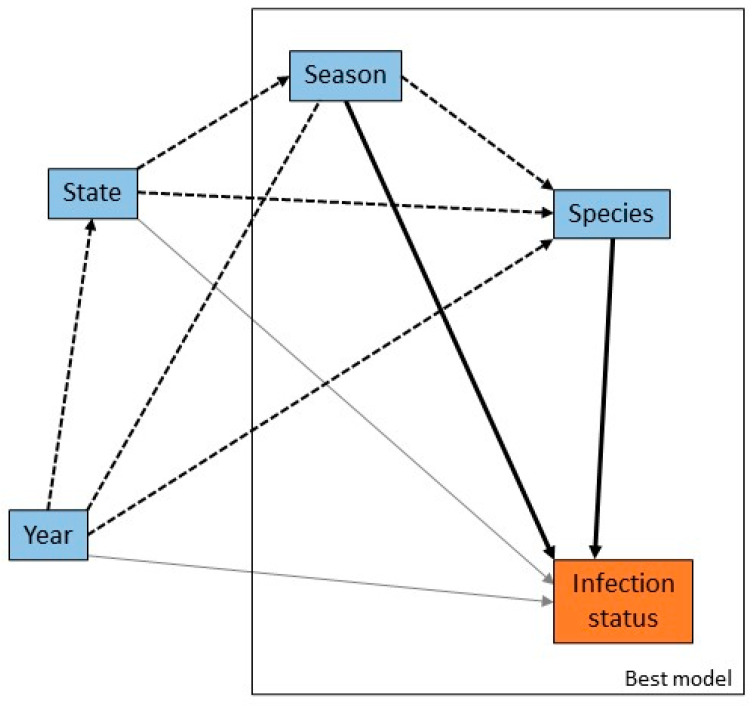
Causal web of association between infection risk factors and ABLV infection status. Heavy black arrows are significant univariable associations between the risk factors and infection status, dotted arrows are significant associations between pairs of risk factors, and grey arrows are plausible causal associations that were not statistically significant in our dataset. The rectangle encloses the suite of variables that produced the best multivariable model.

There were significant univariable associations between ABLV infection status and season and species but not state, and an association bordering on significant between ABLV infection status and year (Figure 3). The likelihood of infection was greater in the austral summer and autumn, in the years 2014 and 2015, and for LRFF and SFF.

The multivariable model that best explained the data (AIC 697) and fitted adequately (likelihood ratio test *p* < 0.01; Hosmer-Lemeshow goodness-of-fit test *p* = 0.90) included only season and species (Table 3). The two explanatory variables season and species are correlated (*p* < 0.01) and season is likely to be a confounder in the relationship between species and ABLV status due to differences in breeding cycles in different species. The odds ratios calculated from the multivariable model are lower than those calculated for the two univariable models, with the effect of spring reduced from borderline significant to not significant. Adding an interaction term for season and species did not improve the fit of the model.

### 3.2. Circumstances and Clinical Signs in Pteropid Bats

**Figure 4:**
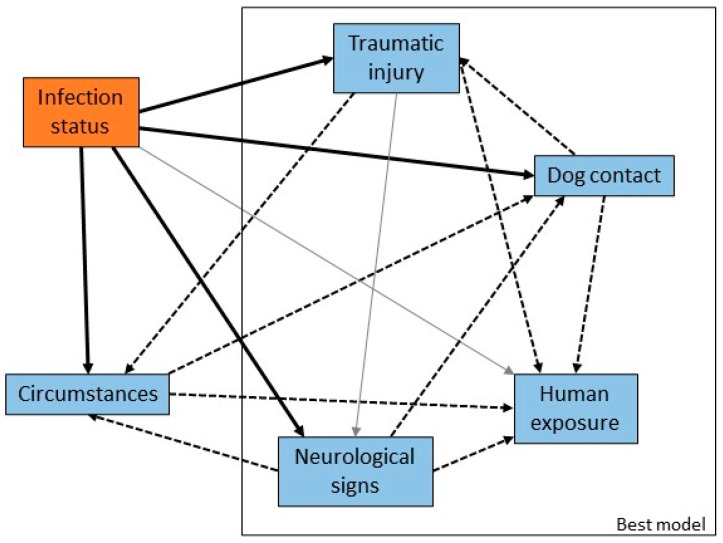
Causal web of association between presentation or clinical signs and ABLV infection status. Heavy black arrows are significant univariable associations between the presentation or clinical sign and infection status, dotted arrows are significant associations between pairs of variables, and grey arrows are plausible causal associations that were not statistically significant in our dataset. The rectangle encloses the suite of variables that produced the best multivariable model.

Variables were created to capture information about the bat at the time of rescue: the circumstances in which the bat was found (such as grounded or orphaned), the presence of traumatic injury or neurological signs, and human or dog contact. There were significant univariable associations between infection status and circumstances, neurological signs, traumatic injury and dog contact, and a borderline significant association with human contact (Figure 4). The odds of infection were higher in bats with neurological signs and those that had been in contact with humans relative to those that had not, and lower in bats with traumatic injury or dog contact. There were statistically significant correlations between explanatory variables related to presentation and clinical signs of infection, reflecting both the potential for confounding and overlap between categories of circumstances and the other variables.

The most parsimonious multivariable model (AIC 589) with adequate model fit (likelihood ratio test *p* < 0.01; Hosmer-Lemeshow goodness-of-fit test *p* = 0.87) included presence of neurological signs, presence of trauma, human contact and dog contact (Table 4). The odds ratios estimated in the multivariable model were smaller for neurological signs and traumatic injury, and larger for human and dog contact, relative to the corresponding single variable models. These variables are all potential confounders for each other (as indicated in Figure 4), so these odds ratios should be considered as adjusted for the effects of the other variables.

Circumstances alone produced a model with a larger AIC (666) but still fitted adequately (likelihood ratio test *p* < 0.01, Hosmer-Lemeshow goodness-of-fit test *p* = 1) and contains some useful information (Table 5). Bats with abnormal behaviour or those that were ‘grounded’ had greater infection risk relative to bats for which circumstances were not recorded (unspecified). Those found tangled in fruit netting or fences, and those involved in pet contact, had lower odds of infection. Those found dead or submitted from mass mortality or morbidity events did not have a significantly different odds of infection compared to bats for which circumstances were not recorded (unspecified).

### 3.3. Risk Factors for Human Contact with Flying-Foxes

**Figure 5:**
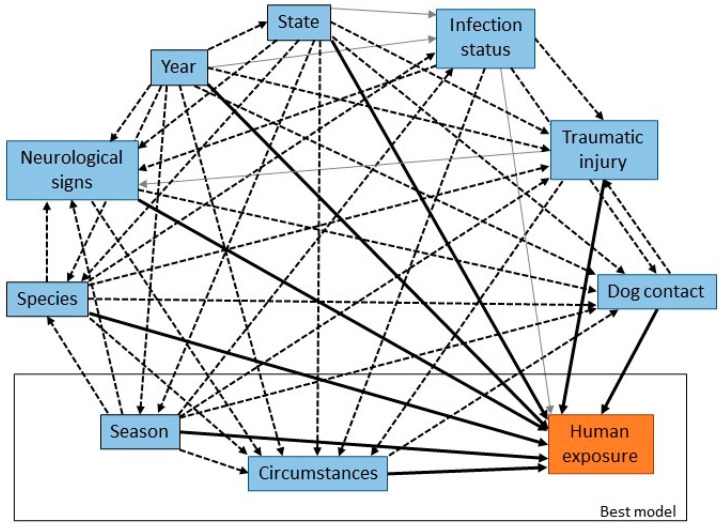
Causal web of association between risk factors and human contact. Heavy black arrows are significant univariable associations between risk factors and infection status, dotted arrows are significant associations between pairs of risk factors, and grey arrows are plausible causal associations that were not statistically significant in our dataset. The rectangle encloses the suite of variables that produced the best multivariable model.

There were significant univariable associations between human contact and year, season, species, state, circumstances, neurological signs, traumatic injury, and dog contact, and a borderline significant association with ABLV infection status (Figure 5). There was increased likelihood of human contact in 2010 and 2012; all seasons relative to winter; GHFF and LRFF; New South Wales; bats found in circumstances of abnormal behaviour, entanglement, grounded or orphaned; bats with traumatic injury; and bats that tested positive for ABLV. There was decreased likelihood of human contact in bats with neurological signs and those that were involved in dog contact.

Circumstances and season alone produced the most parsimonious model (AIC 1441) with adequate model fit (likelihood ratio test *p* < 0.01; Hosmer-Lemeshow goodness-of-fit test *p* = 0.58) (Table 6). For ease of interpretation, the referent category was changed to ‘pet contact’, which was the category with the lowest odds of human exposure. The odds ratios estimated in the multivariable model were generally greater for circumstances and lower for season relative to the respective single variable models.

Animals for which no information on circumstances was recorded were around 65 times more likely to have had a contact with a person. Similarly, there was greater likelihood of human contact among bats without demographic information available (OR 11.43 (8.36–16.03) for sex and 11.68 (8.82–15.78) for age).

### 3.4. Time Series Analysis

**Figure 6:**
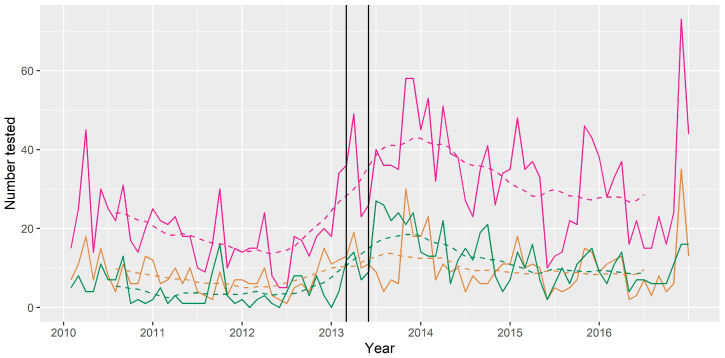
Number of bats tested for ABLV per month (trend—broken line) 2010–2016. Pink = all bats tested; green = bats with dog contact; tan = bats with human contact. Year ticks represent 1st January (mid-summer). The two vertical black lines represent the timing of the human case and the horse cases, which both received media attention.

The median number of bats tested per month for the period 2010 to 2016 was 24, with a minimum of 5 and a maximum of 73. The trend component showed a slightly decreasing trend between 2010 and 2012, followed by an increasing trend with a peak in late 2013, and then a plateau in later years of the series. The peak in number tested per month in late 2013 occurred several months after the human and horse cases, however the trend had already begun to increase before these cases occurred (Figure 6). The seasonal component showed peaks in number of bats tested in November, January and March, and a decrease in May, June and July.

The median monthly prevalence of ABLV among tested bats for the period 2010 to 2016 was 0.03, with a minimum of 0 and a maximum of 0.2. The trend component of the decomposed time series showed a generally higher prevalence in the latter half of the series. The seasonal component of the monthly prevalence showed an increase in April, May, October and December, and a decrease in June and July.

Among the three most commonly tested flying-fox species, BFF and GHFF accounted for the greatest numbers of bats tested per month (min = 0; max = 26; median = 9 for BFF and min = 0; max = 37; median = 5 for GHFF). LRFF were less frequently tested (min = 0; max = 13; median = 2). For all three species the minimum and median monthly prevalence amongst tested bats was 0.

**Figure 7:**
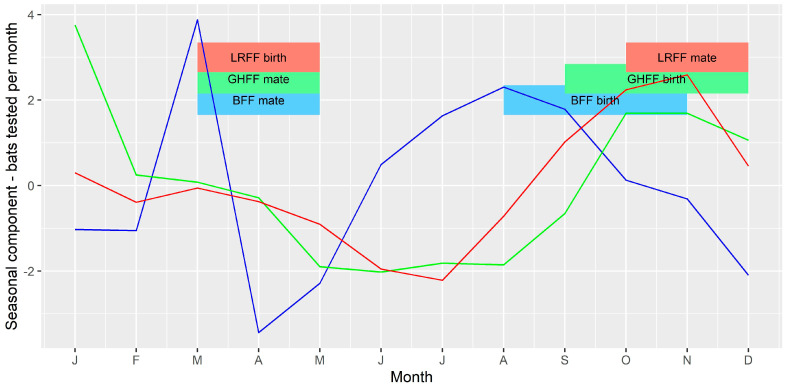
Seasonal component of time series of bats tested per month. The vertical axis represents the expected increase or decrease in number of submissions in each month. Blue = BFF; green = GHFF; red = LRFF.

**Figure 8:**
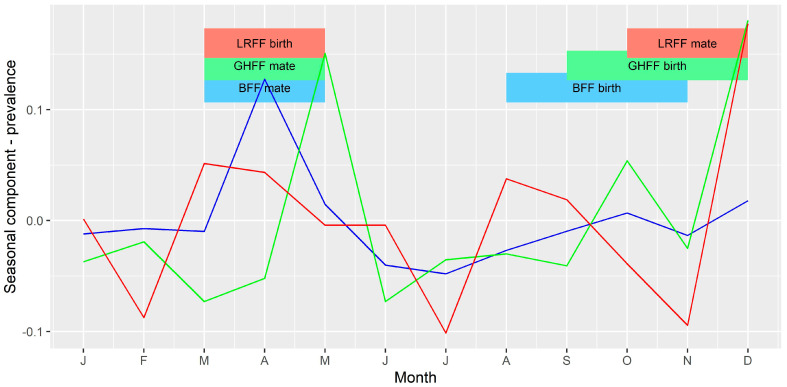
Seasonal component of time series of ABLV prevalence per month. The vertical axis represents the expected increase or decrease in prevalence (as a proportion) in each month. Blue = BFF; green = GHFF; red = LRFF.

The seasonal component of the time series of bats tested per month (Figure 7) and monthly prevalence among bats tested (Figure 8) for each of the three species were compared to the timing of mating and birthing periods. There appeared to be a slight peak in number of bats tested per month just prior to mating for BFF and LRFF but otherwise no clear relationship between number of bats tested and the timing of mating or birthing. There appeared to be peaks in prevalence shortly after the mating and birthing periods for GHFF, and peaks late in the mating period for BFF and LRFF.

## 4. Discussion

In order to effectively mitigate the public health risks due to ABLV, we must understand both disease dynamics in the reservoir host and human behaviours that affect exposure. The results of our data analysis provide some new insights, as well as evidence to support the findings of previous research. In some cases, the new findings may prove to be anomalies resulting from biases in the data, as discussed below.

LRFF in our dataset were more likely to be infected with ABLV than other flying-fox species (using black flying-foxes as the referent group—Table 3), which is consistent with previous research using passive surveillance data [6,8]. Ecological factors including high roosting density, large-scale migration and an extensive geographic range that overlaps with the other species may lead to increased transmission [33] and a genuinely greater infection risk for this species. The finding that spectacled flying-foxes are also more likely to be infected than black flying-foxes has not been observed previously. This species has a limited range compared with other flying-fox species in Australia, being confined to coastal areas of Far North Queensland. Given the small numbers submitted for testing, the finding may be unreliable and influenced by factors relating to the surveillance system.

Similarly, our finding of seasonal differences in the likelihood of infection (Table 3, Figure 8) is at least partially in agreement with those of Field [33] who found that flying-foxes submitted in the first two quarters (i.e., January to June) were around twice as likely to be FAT positive than the referent fourth quarter (October–December). However, Barrett (2004) [8] did not find a seasonal or reproductive season effect in infection prevalence among bats submitted for testing via a passive surveillance system. Three of the Australian *Pteropus* spp., black, spectacled and grey-headed flying-foxes, have a similar reproductive cycle with mating in autumn (March–May) and birthing in spring (September–November) [31,32]. However, little red flying-foxes mate in late spring and give birth in early autumn [31,32]. Studies investigating the ecology of lyssaviruses in other parts of the world have found associations between lyssavirus infection prevalence or seroprevalence and breeding cycles in bat hosts [34,35,36], so it is plausible that these reproductive periods represent times of increased transmission among flying-foxes in Australia. These are also periods of increased likelihood of bat contact for people (Table 6) [24], suggesting that the risk of exposure to infection is greater at these times of year. This supports the current practice of public health agencies issuing public health messaging on ABLV risk around bat breeding seasons.

Similar to previous studies and in common with other lyssaviruses, overt neurological signs were associated with infection status (Table 4) in our study [8]. Two common circumstances of rescued bats that are associated with ABLV infection are ‘grounded’ and unusual behaviour (Table 5). These circumstances are recognised anecdotally and have been described in ABLV-positive bats previously [8], and also in bats affected by rabies lyssavirus in North America [35]. In our data, grounded bats were often described as ‘found on the ground’ or ‘unable to fly’ or both and were significantly associated with ABLV infection. Presentation of a bat by a member of the public using these or similar phrases should immediately raise suspicion of ABLV infection. The abnormal behaviour exhibited by bats was more varied and included unusual vocalisation, self-trauma, and unprovoked aggression towards a person or other animal. These behaviours were significantly associated with ABLV infection, and hence presentation of bats exhibiting these and similar behaviours should also raise suspicions.

Bats found tangled in fences and fruit netting had a lower likelihood of being infected with ABLV, although there was still risk (Table 5). This is consistent with the findings of Barrett (2004) [8]. Bats found under these circumstances were also more likely to have been involved in a human contact (Table 6), and this is one of the most common reasons for people presenting to public health units after bat contact [24]. Despite the lesser prevalence in bats found in this way, the greater frequency of human contact makes this an important pathway by which humans can potentially become infected with the virus. This circumstance also consumes public health resources and post-exposure prophylactic treatments. Prevention of entanglement of bats through promotion of wildlife-safe netting and fencing, in addition to the animal welfare and conservation outcomes, is an effective method of reducing ABLV exposure risk and public health expenditure [37,38].

Some of the unexpected findings of this analysis probably reflect the shortcomings of the passive surveillance system. Because the data are opportunistically collected there are inherent biases, some of which are well understood, and others that are not. The biases arise at a number of points in the surveillance system: (1) bat biology and ecology affects whether bats are observed by people e.g., species distribution, behaviour, and body size; (2) disease ecology affects which bats become infected and whether they can be observed by people when they are clinically affected e.g., when and where they are, and the extent to which clinical signs of infection are apparent; (3) public awareness affects the level of knowledge of the disease, the appropriate methods for diagnosis or the need for medical advice in the event of bat contact; (4) access to diagnostic services affects which bats are tested for ABLV e.g., remoteness of the location, state/territory policy on bat testing; and (5) submitter knowledge and motivation affects the information available about the bat e.g., level of demographic data, history and clinical information recorded. The dataset also represents only a subset of events where a bat comes into contact with a person or pet; in the majority of cases bats are not tested because they cannot be safely captured, or a decision is made not to euthanise a healthy bat for the purpose of testing. Nonetheless, the main reason for which individual bats are tested is to assess the risk of a person or domestic animal being exposed to ABLV from contact with the bat.

One example of the biases in our data is the greater likelihood of demographic data having been recorded for bats that tested positive, and the reduced likelihood for bats that had been involved in human contact. The reasons for the bias are not known but may relate to the expertise and motivation of the submitter and the reason for testing the bat. For bats involved in a human exposure, the focus of information collection is likely to be the human patient rather than the bat. One finding not consistent with previous research is the lack of difference in infection risk between juvenile and adult bats, whereas pups have previously been found less likely to be infected [33]. A possible explanation is that carers, who generally hope to raise and release juvenile bats in their care, selectively submit bats with clinical signs consistent with ABLV and therefore a greater likelihood of being infected. More recently small clusters of disease have been observed in dependent young [39,40] so it is also possible that cases in juveniles in creches have not been detected in the past. Additionally, a modelling study by Hayman and colleagues (2018) [41] suggested that juvenile straw-coloured fruit-bats (*Eidolon helvum*) may be more likely to be infected with Lagos bat virus than adults. It is clear that young bats do pose a risk of transmission of lyssaviruses to people and should be handled as if they are potentially infected. Further studies would be required to determine whether the risk is similar to adult bats, however these studies would not affect the public health advice to treat all ages of bats as potentially infected.

Infection risk in bats that have had contact with humans increased after controlling for neurological signs in a multivariable model (Table 4). Bats with reported neurological signs were also less likely to have been involved in human contact. One potential explanation for these findings is underreporting of neurological signs in bats that were involved in human contact, similar to the suspected reporting bias for demographic information. It is also possible that public health messaging has been somewhat successful and those that handle bats regularly are particularly cautious when handling bats with recognisable neurological signs. Alternatively, neurological dysfunction may compromise, rather than enhance, the ability of clinically affected bats to bite or scratch. Nevertheless, bats with ABLV, after controlling for those with reported neurological signs, had a higher odds ratio of human contact. The public health risk could be further reduced if we understood why this subset of ABLV positive bats has a higher risk of human contact.

Finally, the infection risk changed over time (Figure 6). This is a plausible finding that could indicate periodic outbreaks associated with long distance movements of bats, or immunological or environmental factors. However, in a passive surveillance system, submission of samples can vary over time in association with significant disease events, media attention or government public information campaigns, and the number and type of samples tested can vary as policies are updated over time. This is particularly true in this system where some of the reasons for bat submission (such as pet contact) are unrelated to clinical signs of ABLV infection. The differing infection risk could simply indicate changes to the number and type of bats tested rather than true changes to infection risk in wild populations. Similar observations have been made in submission of bats for European bat lyssavirus testing in the UK, with increased numbers of submissions following detection of a positive case [42]. The authors hypothesised that the media attention following detection of a positive case likely resulted in increased public awareness, affecting bat submissions in subsequent time periods. Media coverage of the human case of ABLV infection was similarly considered to have caused an increase in reporting of bat exposures in humans in a public health region in Brisbane, Queensland in 2013 [43].

Our analysis has augmented previous research into ABLV in Australian bats, although it has been limited by the biases inherent in passively acquired data. Wildlife surveillance is particularly challenging, and we are fortunate to have a curated dataset available for analysis. Further investigation of the new and unexpected findings would require prospective data collection and research that aimed to limit or account for bias.

The surveillance system for this disease in Australia includes both public and private sector participants from animal health, public health and ecology backgrounds. These participants collaborate nationally via an established One Health initiative, the Bat Health Focus Group, to share information and ensure collation of data. Similar collaboration at an operational level to standardise collection of data and information could help to reduce potential biases. We acknowledge that the primary role of the surveillance system is to manage the risk of human and domestic animal health (rather than data collection for post-hoc analysis), however the dataset as a secondary output is clearly useful to inform risk management at a broader level. The data would be more valuable with increased harmonisation of policies and processes for bat testing, data collection and reporting, between the states and territories, and between public health and animal health authorities. Operationally this may lead to a reduction of disease risk by, for example, predicting times and circumstances of higher risk to allow more targeted responses. Continued collaboration across institutions and health sectors will not only assist in management of ABLV risk, but also in responses to other zoonotic diseases in Australia.

Despite the limitations of passive surveillance data, they can be a valuable source of information for wildlife diseases, particularly given that wildlife tends to be less closely observed than domestic animals. Routine surveillance for known diseases in bats and other wildlife can also lead to identification of novel agents that may pose risks to biodiversity, public health or domestic animal health e.g., [44] For zoonotic pathogens such as ABLV, it is important to make use of the data to better understand public health risks. This study used surveillance data to better inform public health authorities attempting to minimise risks to people, gain insight into the functioning of the surveillance system and make recommendations to strengthen data collection to improve interpretation of future ABLV surveillance data.

## Figures and Tables

**Table 1:** Variables used for this analysis.

Variable Name	Encodes	Source
Sex	Sex of the tested bat (Male/Female/Unspecified)	Extracted from free text field if recorded.
Age	Age class of the tested bat (Adult/Juvenile/Unspecified)	Extracted from free text field if recorded.
Reproductive status	Reproductive status of the tested bat (Pregnant/Lactating/Unspecified)	Extracted from free text field if recorded.
Season	Austral seasons, aligned to temperate parts of Australia Summer (December to February) Autumn (March to May) Winter (June to August) Spring (September to November)	Determined from event start date recorded in database
State	State or territory where the bat was found	Location information recorded in database
Year	Year in which the bat was found	Extracted from event start date recorded in database
Month	Calendar month in which the bat was found	Extracted from event start date recorded in database (only used for time series analysis).
Species	Bat species	Taxonomic information recorded in database.
Neurological signs	Bat was recorded as having neurological signs, or neurological signs were described in the case history supplied and interpreted as such by the authors (Yes/Unspecified)	Extracted from free text field if recorded.
Traumatic injury	Bat was recorded as having traumatic injury, or injuries were described in the case history supplied (Yes/Unspecified)	Extracted from free text field if recorded.
Reason for testing	Reason why the bat was submitted for ABLV testing. Hierarchy of potential reasons, with up to three of these recorded for an event: (1) human contact (2) pet contact (3) neurological signs (4) other signs Human contact is assigned as the first submission reason even if the contact was only determined later.	Inferred from free text field
Circumstances	The circumstances in which the bat came to human attention (see Table 2). Designed to capture what is reported by the non-technical observer that finds or rescues the bat, so there is no interpretation of observations into clinical signs.	Extracted from free text field if recorded.
Dog contact	Bat was recorded as having contact or possible contact with a dog (Yes/Unspecified)	Extracted from free text field if recorded.
Human contact	Bat had reported contact with a human (Yes/No) *	Extracted from free text field if recorded.
Infection status	Outcome of testing for ABLV	Testing information recorded in database.

* Human contact would be reported if it had occurred, so the comparison category is considered absence of human contact.

**Table 2:** Category definitions for circumstances variable.

Category	Definition
Abnormal behaviour	History included screaming/vocalising, self-trauma, attacking other animals or feeding in daylight hours.
Entanglement	Most commonly tangled in fruit netting or fences, occasionally other entanglements such as fishing line or tinsel.
Grounded	Found in an abnormal location unable to evade capture. History often includes the phrase ‘found on the ground’ or ‘unable to fly’ or both.
Found dead single	Single bat found dead without any known pet contact
Mass mortality/morbidity event	Multiple animals found dead or unwell in proximity at the same time or over a limited time period, including heat stress events
Orphaned	Described as orphaned or found with a deceased mother; young bats in care, assumed to be orphaned; or foetus tested as well as mother
Other	Various forms of misadventure such as electrocution or hit by car; other miscellaneous presentations such as part of a research project, or translocation.
Pet contact	Any actual or possible contact between a bat and pet animal—usually dog or cat—regardless whether initiated by pet or bat and regardless of whether bat was alive or dead at the time of contact
Unspecified	No information on circumstances of find recorded.

**Table 3:** Effects of season and species on infection status.

Variables	Categories	Multivariable Model		Univariable Model	
		OR (95%CI)	*p*	OR (95%CI)	*p*
**Intercept**		0.02 (0.01–0.05)	<0.01		
Season	Autumn	2.30 (1.12–5.09)	0.03	2.64 (1.30–5.80)	0.01
	Spring	1.57 (0.75–3.50)	0.25	1.93 (0.94–4.27)	0.08
	Summer	2.29 (1.13–5.05)	0.03	2.71 (1.35–5.91)	0.01
	Winter	1		1	
Species	Black flying-fox	1		1	
	Grey-headed flying-fox	1.23 (0.72–2.09)	0.45	1.30 (0.76–2.19)	0.34
	Little red flying-fox	3.01 (1.72–5.23)	<0.01	3.28 (1.89–5.64)	<0.01
	Spectacled flying-fox	4.82 (1.06–16.00)	0.02	5.36 (1.19–17.62)	0.01
	Unidentified flying- fox	0.78 (0.26–1.88)	0.62	0.83 (0.28–1.98)	0.70

**Table 4:** Effects of neurological signs, traumatic injury, human and dog contact on infection status.

Variables	Multivariable Model		Univariable Models	
	OR (95%CI)	*p*	OR (95%CI)	*p*
Intercept	0.03 (0.02–0.05)	<0.01		
Neurological signs	13.30 (7.94–22.78)	<0.01	16.67 (10.83–26.04)	<0.01
Traumatic injury	0.24 (0.10–0.51)	<0.01	0.29 (0.12–0.60)	<0.01
Human contact	2.03 (1.22–3.40)	0.01	1.49 (0.99–2.23)	0.06
Dog contact	0.33 (0.14–0.74)	0.01	0.12 (0.05–0.23)	<0.01

**Table 5:** Effects of circumstances on infection status.

Variables	Categories	OR (95%CI)	*p*
Intercept		0.08 (0.05–0.11)	<0.01
Circumstances	Abnormal behaviour	5.44 (2.21–12.57)	<0.01
	Entanglement	0.23 (0.07–0.59)	<0.01
	Grounded	3.53 (2.14–5.89)	<0.01
	Found dead single	0.41 (0.07–1.40)	0.23
	Mass mortality/morbidity event	0.33 (0.02–1.58)	0.28
	Orphaned	1.76 (0.58–4.45)	0.27
	Other	0.98 (0.28–2.58)	0.96
	Pet contact	0.12 (0.04–0.26)	<0.01
	Unspecified	1	

**Table 6:** Effects of circumstances and year on probability of human contact.

Variables	Categories	Multivariable Model		Univariable Models	
		OR (95%CI)	*p*	OR (95%CI)	*p*
**Intercept**		0.04 (0.02–0.06)	<0.01		
Circumstances	Abnormal behaviour	17.02 (7.28–39.07)	<0.01	16.33 (7.02–37.33)	<0.01
	Entanglement	45.10 (28.23–74.56)	<0.01	43.86 (27.65–72.00)	<0.01
	Grounded	7.05 (4.14–12.17)	<0.01	6.60 (3.90–11.34)	<0.01
	Found dead single	1.60 (0.46–4.28)	0.40	1.63 (0.47–4.37)	0.38
	Mass mortality/morbidity event	1.31 (0.21–4.64)	0.72	1.29 (0.20–4.54)	0.74
	Orphaned	12.64 (5.87–26.82)	<0.01	14.13 (6.62–29.76)	<0.01
	Other	15.48 (7.88–30.42)	<0.01	14.70 (7.51–28.76)	<0.01
	Unspecified	68.44 (44.37–109.70)	<0.01	64.92 (42.36–103.41)	<0.01
	Pet contact	1		1	
Year	Winter	1		1	
	Autumn	0.81 (0.54–1.23)	0.32	1.53 (1.12–2.10)	0.01
	Spring	1.41 (0.95–2.10)	0.09	1.91 (1.42–2.57)	<0.01
	Summer	0.94 (0.63–1.41)	0.76	1.82 (1.34–2.47)	<0.01

## Data Availability

Restrictions apply to the availability of these data. Data was obtained from the eWHIS database managed by Wildlife Health Australia, with data ownership retained by the numerous institutions and individuals that submit the data. Data are available from the authors with the permission of the submitters.
